# Can Early Thyroid Profiling Help Avert Spontaneous Abortions/Early Pregnancy Loss: A Retrospective Study

**DOI:** 10.7759/cureus.18003

**Published:** 2021-09-15

**Authors:** Meenakshi Sundaram Andra Suryanarayana, Kishore Vellingiri, Saransh Kumar Agarwal N, Bhushan Mohan

**Affiliations:** 1 Biochemistry, PSG Institute of Medical Sciences and Research, Coimbatore, IND; 2 Orthopaedics, Sri Devaraj Urs Academy of Higher Education and Research, Kolar, IND; 3 Medicine and Surgery, PSG Institute of Medical Sciences and Research, Coimbatore, IND

**Keywords:** thyroid profile, pregnancy loss, spontaneous abortions, anti-tpo antibodies, tsh levels

## Abstract

Presence of thyroid autoantibodies in euthyroid women can result in various complications such as miscarriages and pre-eclampsia. Women who are hypothyroid have an increased risk of anaemia, infertility, and preterm birth. Spontaneous miscarriages have been reported in association with women with antithyroid antibodies. This can be utilised as a supplementary marker for the mother's immune system failure. The goal of this study was to compare the thyroid profiles of pregnant women who had a normal delivery to those who had a high-risk obstetric history, and to see if early thyroid profiling can help prevent poor pregnancy outcomes. In conclusion, our analysis has demonstrated that women with abnormal values of T4, T3, anti-thyroid peroxidase (TPO), and TSH were associated with fetal demise when compared to women with normal values of T4, T3, anti-TPO, and TSH. Anti-TPO levels were shown to be elevated in women with a poor obstetric history, making early thyroid profiling improve outcomes in pregnancy. Hypothyroidism with increased TSH and anti-TPO levels may have a negative impact on obstetric history, resulting in the loss of an early pregnancy.

## Introduction

Thyroid peroxidase (TPO) is an enzyme found in the thyroid gland's apical membrane that aids in the conversion of iodine ions (I-) to iodine (I) atoms, a process known as organification. For the formation of thyroxine (T4) and triiodothyronine, these iodine atoms will bind to the tyrosine residues connected to thyroglobulin molecules (T3). TPO plays a supportive role in the formation of the thyroglobulin molecule as well as thyroid hormone coupling events. Anti-TPO antibodies, which belong to the IgG immunoglobulin class, are autoantibodies directed against thyroid peroxidase protein [1]. These antibodies are seen in patients with thyroid dysfunction (hypothyroidism or hyperthyroidism), but there is also evidence of these antibodies in healthy individuals. In Hashimoto's thyroiditis, anti-TPO antibodies can be one of the autoantibodies targeting the gland [2]. Euthyroid women with autoantibodies can cause complications in pregnancy such as repeated miscarriages and pre-eclampsia, according to one study [3]. Euthyroid women are also at a higher risk of anemia, infertility, and preterm deliveries [4]. According to one study, spontaneous miscarriages are linked to a higher incidence of antithyroid antibodies [5]. This can be used as a secondary marker for immune system failure in the mother [6]. The goal of this study was to compare the thyroid profiles of pregnant women who had a normal delivery with those who had a complicated obstetric history, and to see if early thyroid profiling can help prevent bad pregnancy outcomes.

## Materials and methods

From December 2019 to December 2020, investigators at PSG Institute of Medical Sciences and Research in Coimbatore conducted a retrospective analysis. After receiving approval from the institutional ethics committee, we reviewed data from 164 patients, including 81 patients with thyroid problems and 83 healthy pregnant women. Age, BMI, anti-TPO levels, T3, T4 levels, mode of delivery, and thyroid medication were all taken into consideration. The information obtained on chart review of the 164 patients was documented on a password-protected online tool and accessed by the investigators only. None of the patients protected health information (PHI) were documented as per the institutional Health Insurance Portability and Accountability Act (HIPAA) policy. The mean, standard median, and confidence interval were used to express all quantitative data. A student t-test was used to quantify the magnitude of the mean difference between groups, and a p-value of 0.05 was considered statistically significant.

## Results

**Figure 1:**
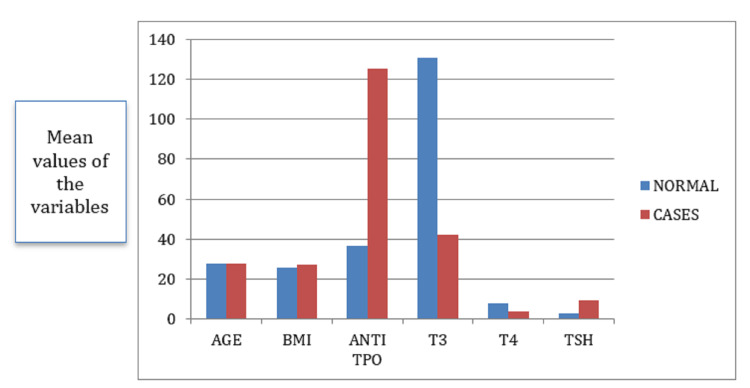
The mean values of the variables assessed between the groups are shown in a bar diagram. BMI: body mass index; anti-TPO: anti-thyroid peroxidase; T3: thyroxine; T4: free or total triiodothyronine; TSH: thyroid-stimulating hormone.

The mean age group in normal individuals was 27.67 years old, while thyroid patients were 27.43 years old. In normal and thyroid disorders, the mean BMI was 25.49 and 27.18, respectively. Anti-TPO levels were 36.4 and 125.9 in normal and thyroid individuals, respectively. T3 levels were 130.7 and 42.06 in the normal and thyroid groups, respectively. T4 levels were 7.941 and 3.638 in the normal and thyroid groups, respectively. TSH levels were 2.897 and 9.405 in the normal and thyroid groups, respectively. The variables' p values are listed in Table 1. Except for the age, all of the p values were statistically significant. Table 2 shows age and BMI are statistically significant at the significance level of 0.05 using logistic regression. We can deduce that variations in the "independent variable" (anti-TPO, T3, T4, and TSH) are linked to changes in the event's probability. Age and BMI were factored into all models. When the odds ratio exceeds one, the event becomes more likely as the value of the predictor or independent variable rises. The mean values of the variables assessed between the groups are shown in a bar diagram below in Figure 1.

**Table 1:** Between the groups, the mean, standard deviation and p values of the variables. BMI: body mass index; anti-TPO: anti-thyroid peroxidase; T3: thyroxine; T4: free or total triiodothyronine; TSH: thyroid-stimulating hormone.

Variables		Normal	Cases	p-value
		n = 81	n = 83	
Age	Mean	27.67	27.43	0.8407
	Standard deviation	5.435	5.345	
BMI	Mean	25.49	27.18	0.006
	Standard deviation	4.053	3.830	
Anti-TPO	Mean	36.40	125.9	<0.0001
	Standard deviation	63.56	99.81	
T3	Mean	130.7	42.06	<0.0001
	Standard deviation	43.85	25.27	
T4	Mean	7.941	3.638	<0.0001
	Standard deviation	3.297	2.937	
TSH	Mean	2.897	9.405	0.0002
	Standard deviation	1.946	15.33	

**Table 2:** For age and BMI, a logistic regression model was developed. OR: odds ratio; CI: confidence interval; anti-TPO: anti-thyroid peroxidase; TSH: thyroid-stimulating hormone.

Regression model adjusted for age and BMI				
Independent variable	OR	95% CI		p-value
Anti-TPO	1.012	1.0037	1.0203	0.0046
T3	0.9349	0.9092	0.9612	<0.0001
T4	0.6804	0.5729	0.8081	<0.0001
TSH	1.1679	1.0276	1.3273	0.0175

## Discussion

Infertility, anemia, and premature birth were all more common in anti-TPO-positive euthyroid females. Anti-TPO screening during pregnancy may aid in the early identification of women at risk [4]. The only autoantibodies that increase the incidence of abortion are antithyroid microsomal antibody and antinuclear antibody [7]. Independent of antiparietal antibodies (APCA) status, women with recurrent spontaneous miscarriages have a greater incidence of antithyroid antibodies (ATA). This could be another indicator of a problem with the maternal immune system's regulation [6]. Anti-peroxidase activity and microsomal antibody titers were found to have a substantial association when assessed using a micro-ELISA (enzyme-linked immunosorbent assay) approach. TPO activity was bound by affinity columns generated from immunoglobulins of MCHA positive microsomal hemagglutination test (+) sera coupled to Reacti-Gel (6X), although the recovery in the unbound fraction was high using control IgG. These findings show that patients with AITD (autoimmune thyroid disease) have antibodies against thyroid peroxidase in their blood, implying a strong relationship between microsomal antigen and thyroid peroxidase [1]. In contrast to the numerous somatic hypermutations found in TPO-specific heavy chains, most TPO-specific light chains, particularly those encoded by J proximal IGLV or IGKV genes, have only limited amino acid replacement, suggesting that a defect in receptor editing can occur during AITD Ab generation. Conserved somatic mutations are the hallmark of the TPO Ab repertoire among the main IGHV1 or IGKV1 TPO Ab. In their study, the authors provided new information about Ab generation against TPO, a key autoantigen implicated in AITD [8]. Finally, because autoantibody (and possibly T cell) responses to TPO are similar to those to TSH receptor and thyroglobulin, manipulating T and B cell responses to TPO could pave the way for immunospecific treatment of autoimmune thyroid disease in general [9]. Antimicrosomal autoantibodies from individuals with Graves' or Hashimoto's thyroiditis were able to bind to isolated TPO and block the mAb binding to purified TPO in a dose-dependent manner. This implies that TPO is the thyroid antigen dubbed the microsomal antigen to date [10]. Low maternal plasma fT4 concentrations in the first trimester of pregnancy may be a significant risk factor for delayed baby development [11]. When hypothyroidism is not limited to the first trimester and worsens as pregnancy develops (as in untreated hypothyroidism), the fetus may be deprived of enough thyroid hormones during subsequent neurological maturation and development, resulting in decreased school performance and IQ [12]. During the first trimester, hypothyroidism, especially subclinical hypothyroidism, is common among north Indian women. To minimize maternal and fetal deleterious effects of hypothyroidism in India, more national research are needed to assess the prevalence and etiology of hypothyroidism [13]. Hypothyroidism was shown to be common in 7% of the population [14], and it has a statistically significant link to recurrent pregnancy losses in the first 20 weeks of pregnancy. Thyroid dysfunction should be checked early on in pregnancy. Given the high prevalence of aberrant TSH in pregnancies, universal screening should be considered because poor thyroid function may increase the risk of miscarriage [14]. In all three groups, TSH was highly linked to abortion in the first trimester [15]. Even if goal TSH levels are not met, intensive follow-up and L-T4 medication may enhance pregnancy outcomes [16]. There is a severe shortage of high-quality research in this field, and large-scale randomized trials are desperately needed. Targeted thyroid function testing in pregnancy should be undertaken in all women which can prevent possible adverse outcomes in pregnancy.

## Conclusions

In our study, we encountered that women with abnormal values of T4, T3, anti-TPO, and TSH were associated with fetal demise when compared to women with normal values of T4, T3, Anti-TPO, and TSH. Anti-TPO levels were shown to be elevated in women with a poor obstetric history, making early thyroid profiling improve outcomes in pregnancy. Hypothyroidism with increased TSH and anti-TPO levels may have a negative impact on obstetric history, resulting in the loss of an early pregnancy. Future studies should concentrate on the possible health benefits of detecting thyroid illness and the effect of treatment on pregnancy outcomes in clinical trials.
